# Sardine Inclusion in a Food Waste-Based Substrate for Rearing Black Soldier Fly (*Hermetia illucens*) Larvae: Effects on Growth Performance, Body Composition, and Gut Microbiome

**DOI:** 10.3390/insects16090977

**Published:** 2025-09-19

**Authors:** Seong-Mok Jeong, Byung Hwa Min, Sang Woo Hur, Jinho Bae, Ki Hwan Park, Kang Woong Kim

**Affiliations:** 1 Aquafeed Research Center, National Institute of Fisheries Science, Pohang 37517, Republic of Korea; smjeong1@korea.kr (S.-M.J.); pkmbh@korea.kr (B.H.M.); maverickhur@korea.kr (S.W.H.); jinhobae@korea.kr (J.B.); 2 Entomo Inc., Cheongju 28304, Republic of Korea; pkh@entomo.kr; 3 Subtropical Fisheries Research Institute, National Institute of Fisheries Science, Jeju 63068, Republic of Korea

**Keywords:** *Hermetia illucens* larvae, food waste substrates, sardine, feed ingredient

## Abstract

**Simple Summary:**

This study investigated the feasibility of upcycling surplus sardines as a feed component for black soldier fly larvae (BSFL) to address their recent surge in Korea. Larvae were reared on food waste-based substrates with varying sardine concentrations (0–100%). While high concentrations of sardines (≥50%) significantly inhibited larval growth and biomass, their inclusion effectively enriched the larvae with valuable omega-3 fatty acids (EPA and DHA). High-sardine diets also altered the larval gut microbiome, leading to decreased microbial diversity. In conclusion, using sardines is a viable upcycling strategy, but optimizing the substrate’s nutrient and moisture balance is crucial to enhance larval productivity.

**Abstract:**

The drastic surge in Sardine landings in Korea underscores the urgent need for sustainable upcycling strategies. However, research on the feasibility of using sardine (SD) in food waste (FW)-based substrates during the cultivation of black soldier fly (*Hermetia illucens*) larvae (BSFL) remains limited. Thus, we aimed to investigate the effect of incorporating varying SD contents (0, 25, 50, 75, and 100%), into which 4-day-old (third-instar) larvae weighing approximately 0.02 g were introduced and reared for 12 days in triplicate. SD inclusion in the substrate had a dose-dependent effect on BSFL growth; higher concentrations (≥50%) markedly inhibited key growth indices, including a significant reduction in total biomass (*p* < 0.05). Incorporating SD into the diet dose-dependently enriched the biomass with eicosapentaenoic acid and docosahexaenoic acid while reducing the relative proportions of saturated and monounsaturated fatty acids (*p* < 0.05). Proteobacteria and Firmicutes were the dominant phyla in the intestinal microbiota of BSFL. Further, SD inclusion altered the gut microbial community structure. Increased SD concentration in the diet led to a progressive reduction in unique genera, indicating decreased microbial diversity at higher inclusion levels. Overall, incorporating SD into FW for BSFL cultivation is feasible; however, optimizing substrate composition—particularly moisture and nutrient balance—is necessary to enhance larval growth and productivity.

## 1. Introduction

Global warming is worsening due to the increasing atmospheric levels of greenhouse gases, such as carbon dioxide; this can primarily be attributed to increased fossil fuel consumption driven by industrial development and population growth since the Industrial Revolution. Climate warming is transforming the composition of local marine communities worldwide [1,2]. In Korea, this global trend is exemplified by the replacement of cold-water fish with warm-water species in coastal fisheries [3], leading to a drastic increase in the production of sardines (SDs) from 90 t in 2021 to 48,027 t in 2023 [4]. While sardines are a key marine food source in Korea, a recent drastic surge in their landings poses a pressing challenge regarding the management of sub-grade catches with diminished freshness or commercial value. A substantial portion of this surplus is processed into discarded waste in landfills, creating considerable economic losses and exacerbating environmental burdens. This situation underscores the urgent need for sustainable upcycling strategies.

The global challenge of food waste (FW) critically undermines sustainable development. Annually, the loss and waste of 1.3 billion tons of food—one-third of global production—consumes 1.4 billion hectares of agricultural land and drives significant environmental degradation [5,6]. Conventional waste management strategies are often inadequate. While incineration can reduce disposal costs, it may contribute to air pollution, and landfills pose significant risks such as greenhouse gas emissions, leachate production, and pest proliferation [7]. Even advanced technologies such as anaerobic fermentation and composting are inefficient in fully recycling the rich nutrients present in FW [8,9,10,11,12,13]. Therefore, developing technologies to upcycle FW into high-value products is imperative for promoting a sustainable and environmentally responsible future.

In this context, the black soldier fly (*Hermetia illucens*) has emerged as a highly promising solution for organic waste valorization. Belonging to the order Diptera, this insect’s omnivorous larval stage is particularly adept at efficiently decomposing various organic materials, including food scraps. Furthermore, the adult stage is considered environmentally safe; it is primarily non-feeding, does not bite or sting humans, and is not a known disease vector [14,15,16,17,18]. These characteristics make the Black Soldier Fly an ideal biological agent for sustainable and circular food waste management.

Black soldier fly larvae (BSFL) are gaining attention as a promising feed ingredient due to their rapid growth rate (approximately 14 days) and high nutritional value. Containing approximately 40% protein and 30% fat, BSFL are considered a sustainable resource with the potential to replace conventional fishmeal and fish oil [19]. Traditionally, fishmeal has been an essential protein source in marine aquaculture, valued for its rich content of n-3 highly unsaturated fatty acids (n-3 HUFAs), such as eicosapentaenoic acid (EPA) and docosahexaenoic acid (DHA). However, the unstable supply and price of fishmeal, driven by fluctuating fish catches, has spurred active research into BSFL as an alternative [20,21,22,23]. However, a significant limitation of BSFL is that, unlike fishmeal, their fat consists of over 60% saturated fatty acids while containing only trace amounts of the n-3 HUFAs essential for the growth of many marine fish, such as olive flounder, which have a limited capacity for endogenous synthesis [24,25]. As most marine fish have a limited ability to synthesize n-3 HUFAs de novo, their dietary intake through feed is crucial for optimal growth [26,27,28,29,30,31]. Notably, the fatty acid composition of BSFL can be modulated by their feeding substrate. Therefore, by incorporating n-3 HUFA-rich sardines (SD) into the BSFL diet, the larval fatty acid profile is expected to be effectively enhanced, maximizing their potential as a fishmeal replacement [32]. However, to our knowledge, no study has evaluated the feasibility of using SD in an FW-based substrate during the cultivation process of BSFL.

This study aimed to investigate how incorporating varying SD contents into FW-based substrates affects the growth performance, nutritional profile, and gut microbiome of BSFL and determine the optimal inclusion ratio which maximizes their value as a sustainable feed ingredient.

## 2. Materials and Methods

### 2.1. Experimental Substrate

The FW used in this study was procured from a commercial processing facility in Cheonan, Chungcheongnam-do, Republic of Korea. The specific FW preparation details are proprietary to the manufacturer; however, the material is known to be produced from general post-consumer FW typical of Korean households. SDs, sourced from Korean coastal waters, were transported to the laboratory and homogenized into a paste using a wet grinder before initiation of the experiment. Five experimental substrates were formulated to investigate the response of BSFL to the inclusion of SD. The control substrate (SD0) consisted of 100% FW, while the four treatment diets were created by replacing FW with SD at inclusion levels of 25% (SD25), 50% (SD50), 75% (SD75), and 100% (SD100) on a wet weight basis. No water was added, as the raw ingredients sufficiently provided the moisture content. Each feed was then thoroughly blended using a hand mixer to ensure uniform consistency before being used in the feeding trial. The substrate was prepared in one large batch and stored for the duration of the experiment, with daily rations taken from the stock (Table S1).

### 2.2. Feeding Trial and Experimental Conditions

The rearing experiment was initiated with 4-day-old (third-instar) BSFL with an average initial body weight of approximately 0.02 g and a total length of approximately 4 mm. For each experimental group, 5000 larvae were stocked into three replicate rectangular plastic tanks (550 × 350 × 140 mm), totaling 18 tanks. The feeding trial was conducted for 12 d in a custom-built insect rearing chamber where the larvae were maintained in complete darkness. Throughout the rearing period, temperature and humidity were maintained at 31.3 ± 0.2 °C and 63.9 ± 0.9%, respectively. Substrate was supplied intermittently rather than daily. Over the entire experimental period, this resulted in an average feeding rate of 0.71 kg/tank/feeding day.

### 2.3. Larval Growth Measurement and Sample Processing

After the rearing experiment, the BSFL were fasted for 48 h to ensure gut clearance for accurate growth measurement. Following the fasting period, larvae were harvested and separated from residual substrate using a custom-built separator. The isolated larvae were then washed with tap water and dewatered. The total weight of the larvae from each tank was then recorded as the total larval biomass (TLB).

From these data, the diet reduction (DR), index of growth by time (IGT), and bioconversion rate (BR) were calculated using the following formulae [33,34]:

(1) $$Diet\;reduction\;\left(DR\right)=\{[Initial\;diet\;\left(g\right)\;-\;residue\;\left(g\right)]/initial\;diet\;(g)\}\;\times \;100$$

(2) Index of growth by time (IGT) = total larval biomass (g)/days of trial (d)

(3) Bioconversion rate (BR) = total larval biomass/feed added × 100

In addition, 20 larvae were randomly selected from each experimental tank to measure individual larval length (LL) and larval height (LH) using a Vernier caliper.

The remaining larval biomass from each replicate was subsequently dried for 15 min at approximately 80 °C in an 18 kW microwave to achieve a final moisture content below 6%. These dried larvae were then ground into a homogeneous powder and used to analyze the proximate composition, fatty acid content, and bound amino acid content.

### 2.4. Proximate Composition

Proximate composition analyses were performed according to the methods of the Association of Official Analytical Chemists [35]. Crude protein was analyzed using an FOSS 8400 Automatic Kjeldahl Nitrogen Determinator (FOSS, Hillerød, Denmark), and crude lipid was determined using a Soxtec System 2043 (FOSS). Moisture content was measured after drying in an oven at 135 °C for 2 h. Ash content was determined after incineration in a muffle furnace at 600 °C for 6 h.

### 2.5. Fatty Acid and Amino Acid Composition

Total lipids were extracted using a chloroform and methanol mixture (2:1). Fatty acids were methylated by heating with a 14% BF_3_-methanol solution (Sigma-Aldrich, St. Louis, MO, USA) at 105 °C for 40 min. Fatty acid analysis was performed using a gas chromatograph (Trace 1310; Thermo Scientific, Waltham, MA, USA) equipped with an SPTM-2560 column (100 m × 0.25 mm, Supelco, Bellefonte, PA, USA). A 37-component fatty acid methyl ester mix (PUFA 37 Component FAME Mix, Supelco) served as the standard.

For bound amino acid composition analysis, 20 mL of 6N HCl was added to the sample, which was then vacuum-sealed and subjected to acid hydrolysis in a dry oven at 110 °C for 24 h. The hydrolysate was filtered through a glass filter, and the filtrate was vacuum-concentrated at 55 °C to evaporate hydrochloric acid and water completely. The concentrated sample was adjusted to 25 mL in a volumetric flask with sodium citrate buffer (pH 2.20), filtered through a 0.45 µm membrane filter, and analyzed using an automatic amino acid analyzer (Biochrom 30+; Biochrom Ltd., Cambridge, UK).

### 2.6. Microbiome Analysis

At the end of the feeding trial, five larvae were randomly sampled from each of the three replicate tanks per experimental group (SD0, SD25, and SD100). For each tank, the midguts from the five larvae were dissected and pooled to create a single composite sample. This resulted in three biological replicate samples per experimental group (*n* = 3) for microbiome analysis. DNA was extracted using a DNeasy PowerSoil Kit (Qiagen, Hilden, Germany) according to the manufacturer’s instructions and quantified using Quant-IT PicoGreen (Invitrogen, Carlsbad, CA, USA). Sequencing libraries were prepared following the Illumina 16S Metagenomic Sequencing Library protocols to amplify the V3 and V4 regions. An input of 5 ng genomic DNA was polymerase chain reaction (PCR)-amplified using 5× reaction buffer, 1 mM dNTP mix, 500 nM each of universal forward and reverse PCR primers, and Herculase II Fusion DNA Polymerase (Agilent Technologies, Santa Clara, CA, USA). The first PCR cycling conditions were as follows: initial heat activation at 95 °C for 3 min; 25 cycles of 95 °C for 30 s, 55 °C for 30 s, and 72 °C for 30 s, followed by a final extension at 72 °C for 5 min. The universal primer pair with Illumina adapter overhang sequences used in the first PCR amplification was (V3-F: 5′-CGTCGGCAGCGTCAGATGTGTATAAGAGACAGCCTACGGGNGGCWGCAG-3′) and V4-R: 5′-GTCTCGTGGGCTCGGAGATGTGTATAAGAGACAGGACTACHVGGGTATCTAATCC-3′).

The first PCR product was purified using AMPure beads (Agencourt Bioscience, Beverly, MA, USA). Subsequently, 2 μL of the purified product was used as a template in a second PCR to add index sequences using Nextera XT Indexed Primers under the same cycling conditions as the first PCR, except with 10 cycles instead of 25. The resulting PCR product was also purified using AMPure beads. The final purified product was quantified using qPCR according to the KAPA Library Quantification kit protocol (KAPA Biosystems) and qualified using a TapeStation D1000 ScreenTape (Agilent Technologies, Waldbronn, Germany). Paired-end sequencing (2 × 300 bp) was performed by Macrogen (Seoul, South Korea) using the Illumina MiSeq platform (Illumina, San Diego, CA, USA).

Raw data obtained by performing next-generation sequencing analysis were processed using Quantitative Insights Into Microbial Ecology 2 (QIIME2) v2023.5 (https://qiime2.org/ accessed on 4 October 2023) [36]. Sequences were filtered, denoised, and merged using the Divisive Amplicon Denoising Algorithm 2 (DADA2) pipeline (https://github.com/benjjneb/dada2/ accessed on 4 October 2023), and chimeric sequences were removed. Each read was clustered into amplicon sequence variants and classified based on the SILVA database v138 (https://www.arb-silva.de/ accessed on 4 October 2023). Alpha diversity, beta diversity, and relative abundance of bacterial communities were analyzed using QIIME2 and visualized using QIIME2 View (https://view.qiime2.org/ accessed on 4 October 2023). Common and specifically existing microorganisms among the groups were analyzed using the Venny server (https://bioinfogp.cnb.csic.es/tools/venny/ accessed on 4 October 2023). To ensure comparability across samples, we performed rarefaction at the sequencing depth corresponding to the sample with the minimum number of reads.

### 2.7. Statistical Analyses

SPSS program version 23 (SPSS Inc., Chicago, IL, USA) was used for statistical analyses. Growth performance results were analyzed using one-way analysis of variance (ANOVA), followed by Tukey’s multiple range test, to obtain statistical evidence to determine whether the overall performance of BSFL was significantly (*p* ˂ 0.05) altered in response to the substrate. BSFL quality parameters (proximate composition, fatty acid profile, and bound amino acid profile) and the alpha diversity of intestinal bacterial communities were analyzed using one-way ANOVA or the Bonferroni-corrected Kruskal–Wallis test. Shapiro–Wilk and Levene’s tests were applied to verify whether the normality and homogeneity of variances were met. Beta diversity was analyzed using Jaccard and Bray–Curtis distance metrics, and statistical significance among groups was assessed using PERMANOVA implemented in the beta-group-significance method of QIIME2.

## 3. Results

### 3.1. Nutrient Composition of the Substrate

The proximate compositions, major fatty acids, and essential amino acids of the FW and SD were analyzed (Table 1). SD and FW used as rearing substrates showed distinct nutritional profiles (Table 1). Specifically, SD was characterized by having significantly higher crude protein (15.9%) and crude lipid (15.43%) contents than FW (4.57% and 5.09%, respectively), whereas FW had a higher moisture content (84.81% vs. 69.72%). The fatty acid compositions of FW and SD were also markedly different. While FW was rich in oleic acid (C18:1n-9; 32.81%) and linoleic acid (C18:2n-6; 24.83%), SD was distinguished by its exceptionally high levels of the marine-derived omega-3 fatty acids EPA (C20:5n-3; 15.08%) and DHA (C22:6n-3; 8.35%). Moreover, the overall profiles of bound amino acids were similar between the two substrates, although SD contained relatively higher levels of the essential amino acids isoleucine and lysine.

### 3.2. Growth Performance and Substrate Utilization

The 12-day rearing experiment showed a survival rate of 80–89%, with no significant difference observed with the addition of SD to the FW substrate (Table 2). The growth indices of BSFL, specifically TLB and IGT, were not substantially different from those of the SD0 and SD25 groups. However, when SD addition exceeded 50% (SD50), TLB and IGT values were lower than those of SD0. Moreover, as the substrate SD content increased, diet reduction decreased incrementally, with the SD100 experimental group recording the lowest diet reduction at 63%. The length and height of the larvae decreased ≥75% SD.

### 3.3. BSFL Proximate Composition

The proximate composition of BSFL was substantially affected by dietary SD supplementation (Table 3). The crude protein content of BSFL was higher in the SD75 and SD100 experimental groups than in the SD0 experimental group. The crude lipid content was significantly higher in the SD100 experimental group than in the SD50 experimental group, with no significant differences observed in the other experimental groups. Ash content decreased with increasing SD content added to the rearing substrate.

### 3.4. BSFL Fatty Acid Composition

The fatty acid composition of BSFL was influenced by the fatty acid profile of the rearing substrate (Table 4). Notably, C12:0 (lauric acid) was undetectable in the rearing substrate but was present in BSFL. C12:0 is a representative saturated fatty acid in BSFL, with a higher C12:0 content in the SD0 group than in the SD100 group. The content of other saturated fatty acids, excluding C10:0 and C12:0, tended to increase with increasing SD content in the rearing substrate. High C18:2n-6 levels were detected in FW, resulting in a higher C18:2n-6 content in SD0 than in SD100. The inclusion of SD in the substrate led to a dose-dependent increase in the contents of EPA, DHA, and total n-3 polyunsaturated fatty acids (PUFAs). Conversely, the relative proportions of saturated fatty acids (SFAs) and n-6 PUFAs decreased as the SD content increased.

### 3.5. BSFL Amino Acid Profile

Except for valine and threonine, the amino acid profiles of BSFL were similar regardless of SD addition to the rearing substrate (Table 5). Lysine and threonine levels tended to decrease with increasing substrate SD content. The lysine content was significantly higher in the SD25 and SD75 experimental groups than in the SD100 group, with no significant differences observed in the other groups. Threonine levels were higher in the SD25 and SD50 experimental groups than in the SD100 group.

### 3.6. Microbiome

**Figure 1:**
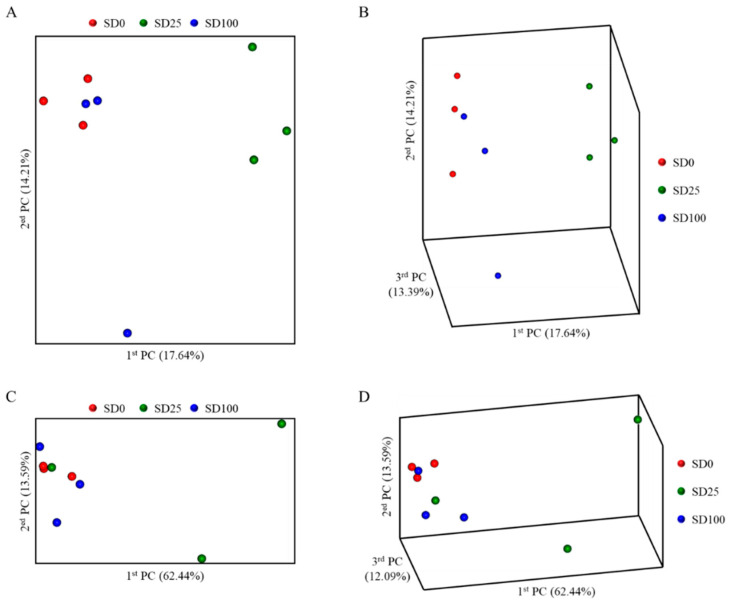
Principal coordinate analysis based on Jaccard (**A**,**B**) and Bray–Curtis (**C**,**D**) distances comparing intestinal bacterial communities of BSFL fed with different substrates.

**Figure 2:**
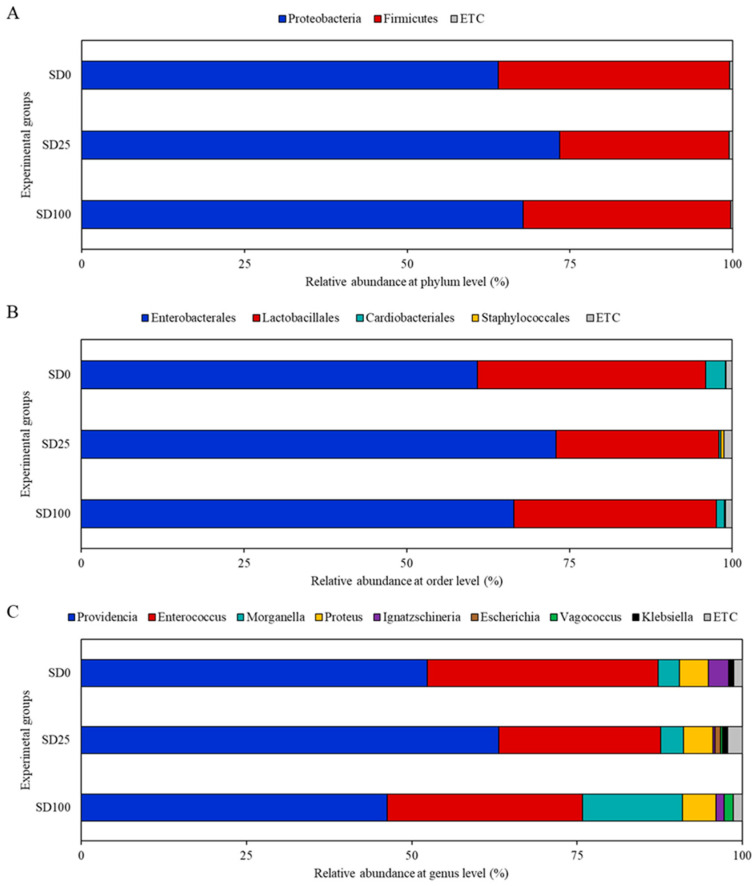
Composition and relative abundance of intestinal bacterial communities of BSFL fed with different substrates at the phylum (**A**), order (**B**), and genus (**C**) levels. ETC includes taxa present at <0.5% relative abundance, such as the phyla Bacteroidota and Actinobacteriota; the orders Bacteroidales and Bacillales; and the genera *Staphylococcus* and *Bacillus*.

**Figure 3:**
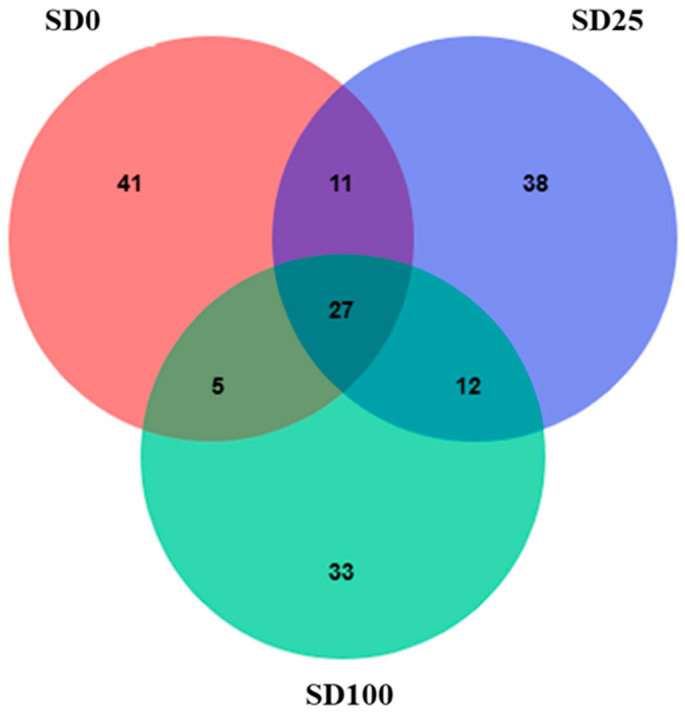
Venn diagram comparing the distribution of genera shared by the gut microbiota of BSFL. The value indicates the common or unique genus number in the applicable groups.

The alpha diversity of the midgut bacterial communities, assessed using multiple metrics (Observed features, Chao1, Faith’s PD, Shannon, and Evenness), showed no significant differences among the experimental groups (*p* > 0.05; Table S2). The bacterial community of the SD100 experimental group clustered closely with that of the control group, indicating similar microbial compositions. Notably, despite the complete replacement of FW with SD as the main ingredient in the diet, no clear separation was observed between the two groups in the PCoA plot (Figure 1). In contrast, the SD25 group was positioned on the right of the PCoA plot, forming a distinct cluster separate from the control and SD100 groups. At the phylum level, the distribution of intestinal microbes in BSFL was compared and analyzed, revealing that Proteobacteria and Firmicutes were the dominant phyla. The relative abundance of Proteobacteria was the highest in the SD25 group (72.3%), followed by in the SD100 (67.0%) and SD0 (62.11%) groups. The abundance of Firmicutes was the highest in the SD0 group (37.5%), followed by in the SD100 (32.9%) and SD25 (27.3%) groups. Additionally, Actinobacteria were present in minimal amounts across all groups, with relative abundances of 0.28, 0.29, and 0.04% in the SD0, SD25, and SD100, respectively (Figure 2). Figure 3 illustrates Venn diagram analysis of the intestinal microbiota across various experimental groups. In the SD0 group, 84 genera were identified, whereas the SD25 and SD100 groups had 88 and 77 genera, respectively. All groups had 27 genera in common. The SD0 group exhibited the highest number of unique genera (41), followed by the SD25 (38 genera) and SD100 (33 genera) groups.

## 4. Discussion

To the best of our knowledge, this study presents one of the first attempts to evaluate the feasibility of using SD in an FW-based substrate during the cultivation process of BSFL. Recently, BSFL has been cultivated using various by-products derived from seafood. Specifically, Pamintuan et al. [37] demonstrated that using by-products from milkfish as a substrate resulted in a higher growth rate of BSFL than that using standard chick feed or a mixture of vegetable waste. In contrast, compared to using chick feed, the use of by-products that include tissues and shells from mitigation mussels (*Mytilus edulis*), as well as heads, appendages, and exoskeletons of shrimp (*Pandalus borealis*) as substrates, results in a substantially lower growth performance of BSFL [38]. Specific seafood by-products may be more suitable for BSFL cultivation under certain conditions. BSFL prefer carbohydrates as an energy source; in the absence of carbohydrates, proteins or lipids serve as alternative energy sources. However, using these alternatives can reduce energy efficiency relative to that when using carbohydrates [38,39,40]. The optimal protein-to-carbohydrate ratio of BSFL substrates is between 1:1 and 1:3 [38,41].

The decrease in growth with increasing SD content in this study may be attributed to an excessively low protein-to-carbohydrate ratio. FW, wherein carbohydrates constitute approximately 65% of the total solids, can serve as an ideal source of carbohydrates for BSFL [42]. The moisture contents of the FW and SD used were 84% and 66%, respectively, resulting in a gradual decrease in moisture content in the substrate with an increase in the proportion of SD. High substrate moisture content is related to the scraping mechanism of BSFL as they feed. Increasing the substrate moisture content can soften the substrate, facilitating easy ingestion by BSFL [43,44,45,46]. Therefore, maintaining high moisture content in an FW substrate can benefit BSFL productivity.

Extreme nutrient compositions in the substrate can also impair productivity. Spranghers et al. [24] reported that using restaurant waste, which has a high ether extract content, lowers growth efficiency relative to that using vegetable waste. In the current study, the increase in SD content led to a maximum increase in crude protein and crude lipid content of 44% and 43% on a dry matter basis, which could have inhibited larval growth. Research by Leroi and Joffraud [47] and Hungerford [48] indicates that fish waste decomposes rapidly under natural conditions, potentially producing toxic substances. Aquaculture waste can form an oil layer in the substrate, which reduces oxygen supply, making respiration difficult for larvae and negatively impacting their survival rate [49]. Although the increased SD content did not alter larval survival rates considerably in the current study, forming an oil layer from SD in the substrate may have interfered with the larvae’s productivity. Therefore, a mixed diet of FW (or kitchen waste) and SD may allow for the normal cultivation of BSFL. Further research is imperative to establish the detailed substrate composition for optimal growth.

In this study, the general BSFL composition varied across the experimental groups. All larvae were harvested on day 14, and remarkable substrate-specific variations in size and developmental stages were observed. Specifically, larvae cultured in the S0 and S25 substrates appeared darker than those cultured in high SD substrates and reached the pre-pupal stage, whereas those cultured in the S100 substrate were lighter, suggesting that they were at a different developmental stage [50]. Differences in the general BSFL composition depend on their developmental stages [51]. BSFL are known for their remarkable ability to convert carbohydrates into lipids [24,45,52]. These larvae efficiently synthesize acetyl-CoA from fatty acids, such as C12:0, C14:0, C16:0, and C18:0, and subsequently convert them into triglycerides. This process suggests that the substrate’s carbohydrate and crude lipid content can directly influence lipid accumulation in the larvae [53,54]. Larger BSFL typically accumulate more fat, whereas protein accumulation is lower than that in smaller BSFL [24,55,56]. However, in this study, the crude lipid content of the larvae did not differ significantly based on size or substrate lipid content. Thus, lipid accumulation in larvae may be influenced by other factors.

Lauric acid was not detected in the rearing substrate of *Hermetia illucens* larvae, but was identified as a major fatty acid within the larvae. Gull-Gerrero et al. [57] reported that these larvae can bioconvert carbohydrates into lipids. Consequently, it was suggested that *Hermetia illucens* larvae can biosynthesize lauric acid using carbohydrates. Furthermore, larvae in advanced development stages accumulate high proportions of SFAs and lauric acid [24,51]. According to Ewald et al. [50], larval weight correlates positively with the percentage of lauric acid and total SFAs in the body. However, it correlates negatively with the content of monounsaturated fatty acids and PUFAs. As a medium-chain triglyceride, lauric acid is efficiently absorbed, digested, and subjected to β-oxidation, making it a viable energy source for *Hermetia illucens* [32,58]. However, due to the high content of lauric acid and SFAs in *Hermetia illucens*, which remain solid at room temperature, their practical application as substrate ingredients or supplements requires additional processing, such as heating. Therefore, further research is necessary to address these challenges.

In this study, the oleic acid (C18:1n-9) content within the substrates differed substantially, with 32.81% in FW and 11.81% in SD. However, the BSFL demonstrated similar oleic acid levels. BSFL may possess the stearoyl Co-A desaturase enzyme (Δ9-desaturase), which can add a cis-9 double bond to saturated and unsaturated fatty acids with carbon chain lengths from 12 to 19 atoms [59,60,61,62]. With an increase in SD content leading to a decrease in the substrate C18:1n-9 content, the larvae continued to increase the proportion of SFAs, C14:0, C16:0, and C18:0. Through the action of Δ9-desaturase, a considerable proportion of C18:1n-9 was produced as unsaturated fat [63]. This mechanism could explain why BSFL fed on pomaces from two grape varieties with differing oleic acid contents exhibit similar oleic acid levels [64].

In the current study, the combined content of EPA and DHA of the SD used as a rearing substrate was approximately 23.43%, which was substantially higher than that of FW (2.67%). The fatty acid composition of the BSFL, among others, was influenced by the fatty acid composition of the substrate. The EPA + DHA ratio in the S100 experimental group was 5.84%, which was more than eight times higher than that in the S0 experimental group (0.72%). Reportedly, feeding discarded fish by-products to BSFL results in higher n-3 PUFA content than that in the control [32,50].

When commercial broiler substrate is administered to BSFL for 3 d, followed by discarded fish for 12 days, the EPA + DHA content increases from 1.5% (in the absence of fish) to 12.1% [65]. This trend confirms the possibility of bioaccumulating EPA and DHA from the fatty acids provided in the rearing substrate. Moreover, the accumulation efficiency varies depending on the substrate’s fatty acid content and type [66]. Although the EPA content in sardine is twice as high as the DHA content, BSFL in the S25 experimental group showed an approximately 10-fold difference. This trend suggests that while BSFL can efficiently accumulate n-3 PUFA from the rearing substrate, they cannot fully bioassimilate it. Therefore, further research on the fatty acid metabolism and accumulation pathways in BSFL is necessary.

Oonincx and Finke [67] reported that the amino acid composition of BSFL remains unaffected by the substrate used. The amino acid composition and levels observed in the current study were similar to those reported by Eggink et al. [37], with differences observed only in lysine and threonine contents across the experimental groups. Lysine and threonine are typically considered limiting essential amino acids in plant-based protein sources; however, insects are known to contain these amino acids at relatively high levels [24,68,69]. Makkar et al. [70] suggest that insects can match the essential amino acid profile for rearing pigs and broiler chickens. In BSFL, the typical crude protein content based on dry matter ranges from 30% to 50%, which can further increase to over 55% after a defatting process. Therefore, BSFL are considered suitable as a protein and essential amino acid source for the substrate of various species. Further research is needed to elucidate the mechanisms by which the type of substrate influences the accumulation of amino acids in the larvae.

Proteobacteria and Firmicutes are dominant phyla in the gut of black fly larvae reared using pig and chicken manure [11,71]. Including antibiotics in the substrate increases the abundance of Bacteroidetes, which is associated with antibiotic degradation [72,73,74,75,76]. Microorganisms of the genus *Providencia* are known to play an important role in lipid and protein conversion in the gut [72]. The growth rate of the SD25 group was similar to that of the control group, suggesting that *Providencia* was dominant due to efficient protein and lipid utilization. Functionally, *Providencia* has been demonstrated to be a dominant member of the BSFL gut microbiota, actively promoting key developmental processes including larval weight gain, progression to prepupae, and successful eclosion [77,78]. Interestingly, while a previous study reported a higher relative abundance of Providencia in larvae fed specific plant-based substrates [78], our findings show a contrasting result: Providencia was most dominant in the SD25 group, which was characterized by a high content of animal protein. This suggests that Providencia may not be limited to a specific nutrient source but could be a keystone species that promotes larval growth under diverse substrate conditions. In contrast, Morganella, which was highly abundant in the SD100 group, is known to induce immune responses [78,79,80]. Therefore, the growth inhibition observed in this group could be associated with an unnecessary activation of the immune system by Morganella, although further research is required to establish a clear causal relationship. However, further research is needed on the microbiome concerning the rearing substrate’s raw material, form, and nutrient content. Substantial variations are evident in the gut microbiome of BSFL, depending on the type and nutrient content of the substrate. Therefore, future in-depth studies are imperative to elucidate precisely how diverse inputs, such as specific organic wastes and feed ingredients, modulate the assembly and structure of these microbial communities.

## 5. Conclusions

Overall, incorporating SD—a potential raw material for specialized fish feed—into FW-based rearing substrates effectively produced functional BSFL by altering their nutritional composition. This efficacy was evidenced by a significant accumulation of valuable omega-3 fatty acids, such as EPA and DHA, in the larvae. However, a trade-off was observed, as higher SD inclusion decreased growth performance. From a practical standpoint for commercial producers, this presents a critical decision: a high-SD strategy may yield a value-added, omega-3-enriched product commanding a premium price, but the reduced growth rate could lower the total biomass output. Therefore, our recommendation of a 25% replacement of FW with SD represents a bioeconomic sweet spot. This level serves as an optimal strategy to achieve a significant nutritional enhancement without severely compromising the production yield essential for commercial viability. Therefore, to produce insect-based ingredients that are both nutritionally superior and commercially viable, it is necessary to identify an optimal substrate composition that balances rapid larval growth with the fortification of specific fatty acids and cultivation of a beneficial microbiome.

## Figures and Tables

**Table 1:** Proximate (%), major fatty acid (% of total fatty acids), and amino acid composition (% in protein) of FW and SD used as a substrate for BSFL (as basis).

	FW	SD
Proximate composition		
Moisture	84.81	69.72
Crude protein	4.57	15.90
Crude lipid	5.09	15.43
Ash	1.66	3.54
Crude fiber	3.49	0.15
Major fatty acid composition		
C14:0	1.56	8.92
C16:0	20.98	20.89
C18:0	8.43	4.46
C16:1	2.21	11.21
C18:1n-9	32.81	11.81
C18:2n-6	24.83	3.12
C18:3n-3	3.83	1.48
C20:5n-3 (EPA)	0.99	15.08
C22:6n-3 (DHA)	1.68	8.35
Amino acid composition		
Arginine	3.79	3.38
Histidine	1.78	1.86
Isoleucine	3.58	5.22
Leucine	7.12	7.30
Lysine	6.86	8.81
Methionine + Cystine	3.79	4.89
Phenylalanine	3.03	2.50
Threonine	3.64	3.00
Valine	6.59	6.55

BSFL, Black soldier fly larvae; FW, food waste; SD, sardine.

**Table 2:** Growth performance of BSFL reared on FW-based substrates with different SD inclusion levels.

	SR ^1^	DR ^2^	IGT ^3^	LTB ^4^	BR ^5^	LL ^6^	LH ^7^
SD0	89.6 ± 0.9	86.0 ± 0.4 ^a^	93.3 ± 1.0 ^a^	1.12 ± 0.01 ^a^	13.2 ± 0.1 ^a^	28.0 ± 0.5 ^a^	5.88 ± 0.02 ^a^
SD25	85.3 ± 1.1	79.4 ± 0.5 ^b^	88.9 ± 1.1 ^ab^	1.07 ± 0.01 ^ab^	12.5 ± 0.2 ^ab^	26.7 ± 0.3 ^a^	5.82 ± 0.04 ^a^
SD50	82.7 ± 1.1	73.1 ± 1.0 ^c^	86.1 ± 1.1 ^b^	1.03 ± 0.01 ^b^	12.2 ± 0.2 ^b^	27.4 ± 0.2 ^a^	5.92 ± 0.02 ^a^
SD75	80.0 ± 3.1	69.7 ± 1.0 ^d^	66.7 ± 2.5 ^c^	0.80 ± 0.03 ^c^	9.4 ± 0.4 ^c^	23.6 ± 0.2 ^b^	4.88 ± 0.02 ^b^
SD100	84.4 ± 2.9	63.3 ± 0.2 ^e^	42.2 ± 0.6 ^d^	0.51 ± 0.01 ^d^	6.0 ± 0.1 ^d^	20.3 ± 0.2 ^c^	3.77 ± 0.07 ^c^

Values are the mean of triplicate groups and presented as mean ± SE. Different superscript letters (^a^, ^b^, ^c^, ^d^, ^e^) indicate significant differences among treatments (*p* < 0.05). The absence of superscript letters indicates no significant differences among treatments. ^1^ Survival rate (%); ^2^ Diet reduction (%); ^3^ Index of growth by time (g/d); ^4^ Larval total biomass (g); ^5^ Bioconversion rate (%); ^6^ Larval length (mm/larvae); ^7^ Larval height (mm/larvae). BSFL, Black soldier fly larvae; FW, food waste; SD, sardine; SD0, SD25, SD50, SD75, and SD100 represent substrates with 0%, 25%, 50%, 75%, and 100% SD inclusion in FW-based substrates, respectively.

**Table 3:** Proximate Composition (%) of BSFL reared on FW-based substrates with different SD inclusion levels.

	Moisture	Crude Protein	Crude Lipid	Ash
SD0	4.16 ± 0.11	39.6 ± 0.2 ^c^	34.8 ± 0.1 ^ab^	10.22 ± 0.34 ^a^
SD25	4.68 ± 0.20	40.8 ± 0.4 ^bc^	33.1 ± 0.5 ^ab^	9.59 ± 0.11 ^a^
SD50	4.97 ± 0.44	40.6 ± 0.4 ^bc^	31.0 ± 0.3 ^b^	9.79 ± 0.06 ^a^
SD75	5.79 ± 0.06	43.2 ± 0.6 ^a^	32.6 ± 0.6 ^ab^	8.32 ± 0.20 ^b^
SD100	5.63 ± 0.08	41.8 ± 0.2 ^ab^	36.4 ± 0.1 ^a^	5.85 ± 0.03 ^c^

Values are the mean of triplicate groups and presented as mean ± SE. Different superscript letters (^a^, ^b^, ^c^) indicate significant differences among treatments (*p* < 0.05). The absence of superscript letters indicates no significant differences among treatments. BSFL, Black soldier fly larvae; SD, sardine; SD0, SD25, SD50, SD75, and SD100 represent substrates with 0, 25, 50, 75, and 100% SD inclusion in FW-based substrates, respectively.

**Table 4:** The composition of major fatty acids (% of total fatty acids) of BSFL reared on FW-based substrates with different SD inclusion levels.

	SD0	SD25	SD50	SD75	SD100
C10:0	2.49 ± 0.04 ^a^	2.52 ± 0.16 ^a^	2.59 ± 0.13 ^a^	2.12 ± 0.03 ^ab^	1.93 ± 0.10 ^b^
C12:0	51.2 ± 0.2 ^a^	46.3 ± 2.1 ^ab^	44.2 ± 0.2 ^ab^	41.9 ± 1.5 ^ab^	34.8 ± 0.4 ^b^
C14:0	6.16 ± 0.08 ^c^	7.05 ± 0.16 ^b^	7.19 ± 0.09 ^b^	8.06 ± 0.04 ^a^	8.30 ± 0.13 ^a^
C16:0	11.9 ± 0.2 ^b^	13.1 ± 0.4 ^ab^	12.6 ± 0.1 ^ab^	13.7 ± 0.4 ^b^	15.2 ± 0.2 ^a^
C18:0	1.92 ± 0.03 ^b^	2.11 ± 0.04 ^ab^	2.02 ± 0.04 ^ab^	2.24 ± 0.18 ^ab^	2.95 ± 0.12 ^a^
C16:1	1.80 ± 0.15 ^e^	3.82 ± 0.32 ^d^	5.50 ± 0.10 ^c^	7.59 ± 0.22 ^b^	9.81 ± 0.04 ^a^
C18:1n-9	11.9 ± 0.1	11.7 ± 0.4	10.5 ± 0.1	10.7 ± 0.6	11.8 ± 0.4
C18:2n-6	10.4 ± 0.02 ^a^	8.51 ± 0.29 ^ab^	6.99 ± 0.05 ^ab^	5.46 ± 0.18 ^ab^	3.98 ± 0.06 ^b^
C18:3n-3	1.34 ± 0.02	1.01 ± 0.09	1.05 ± 0.01	0.97 ± 0.03	1.01 ± 0.02
C20:5n-3	0.73 ± 0.01 ^d^	2.27 ± 0.15 ^c^	3.32 ± 0.08 ^b^	3.54 ± 0.15 ^b^	4.87 ± 0.16 ^a^
C22:6n-3	n.d. ^b^	0.21 ± 0.02 ^ab^	0.61 ± 0.09 ^ab^	0.39 ± 0.02 ^ab^	0.97 ± 0.11 ^a^
EPA + DHA	0.72 ± 0.01 ^d^	2.48 ± 0.14 ^c^	3.93 ± 0.17 ^b^	3.93 ± 0.16 ^b^	5.84 ± 0.26 ^a^
∑SFA	73.6 ± 0.0 ^a^	71.9 ± 1.5 ^ab^	70.2 ± 0.1 ^ab^	69.9 ± 1.0 ^ab^	65.9 ± 0.6 ^b^
∑MUFA	13.7 ± 0.0 ^b^	15.7 ± 0.8 ^ab^	16.7 ± 0.0 ^ab^	18.5 ± 0.8 ^ab^	22.4 ± 0.4 ^a^
∑n-3 PUFA	2.07 ± 0.03 ^d^	3.50 ± 0.23 ^c^	4.98 ± 0.16 ^b^	4.90 ± 0.19 ^b^	6.85 ± 0.24 ^a^
∑n-6 PUFA	10.39 ± 0.02 ^a^	8.57 ± 0.32 ^ab^	7.24 ± 0.06 ^ab^	5.97 ± 0.11 ^ab^	4.67 ± 0.09 ^b^

Values are the mean of triplicate groups and presented as mean ± SE. Different superscript letters (^a^, ^b^, ^c^, ^d^, ^e^) indicate significant differences among treatments (*p* < 0.05). The absence of superscript letters indicates no significant differences among treatments. BSFL, Black soldier fly larvae; FW, food waste; SD, sardine; n.d., not detected. SD0, SD25, SD50, SD75, and SD100 represent substrates with 0%, 25%, 50%, 75%, and 100% SD inclusion in FW-based substrates, respectively.

**Table 5:** Amino acid composition (% in protein) of BSFL reared on FW-based substrates with different SD inclusion levels.

	SD0	SD25	SD50	SD75	SD100
Arginine	5.23 ± 0.10	5.21 ± 0.11	5.23 ± 0.12	5.26 ± 0.31	5.01 ± 0.03
Histidine	3.61 ± 0.33	3.72 ± 0.08	3.47 ± 0.04	3.97 ± 0.14	3.38 ± 0.10
Isoleucine	4.70 ± 0.04	4.47 ± 0.08	4.69 ± 0.01	4.62 ± 0.08	4.63 ± 0.01
Leucine	7.12 ± 0.11	7.17 ± 0.05	7.26 ± 0.14	7.33 ± 0.10	6.97 ± 0.04
Lysine	6.19 ± 0.03 ^ab^	6.33 ± 0.02 ^a^	6.19 ± 0.09 ^ab^	6.37 ± 0.12 ^a^	5.86 ± 0.06 ^b^
Methionine + Cystine	2.17 ± 0.07	2.06 ± 0.10	2.05 ± 0.24	2.04 ± 0.22	1.73 ± 0.26
Phenylalanine	4.68 ± 0.15	4.29 ± 0.09	4.34 ± 0.08	4.15 ± 0.28	3.92 ± 0.04
Threonine	3.80 ± 0.06 ^ab^	3.92 ± 0.02 ^a^	3.85 ± 0.10 ^a^	3.68 ± 0.09 ^ab^	3.51 ± 0.02 ^b^
Valine	6.65 ± 0.04	6.37 ± 0.03	6.76 ± 0.13	6.67 ± 0.16	6.48 ± 0.01

Values are the mean of triplicate groups and presented as mean ± SE. Different superscript letters (^a^, ^b^) indicate significant differences among treatments (*p* < 0.05). The absence of superscript letters indicates no significant differences among treatments. BSFL, Black soldier fly larvae; FW, food waste; SD, sardine; SD0, SD25, SD50, SD75, and SD100 represent substrates with 0%, 25%, 50%, 75%, and 100% SD inclusion in FW-based substrates, respectively.

## Data Availability

The data that support the findings of this study are available from the corresponding author upon reasonable request.

## Supplementary Materials

The following supporting information can be downloaded at: https://www.mdpi.com/article/10.3390/insects16090977/s1, Table S1: Calculated proximate composition of the experimental substrates (% as fed-basis), Table S2: Alpha diversity of intestinal bacterial communities of Black soldier fly larvae.
