# Supplementary material for: Sardine Inclusion in a Food Waste-Based Substrate for Rearing Black Soldier Fly (Hermetia illucens) Larvae: Effects on Growth Performance, Body Composition, and Gut Microbiome
Source: Insects. 2025 Sep 19;16(9):977. doi: 10.3390/insects16090977 (PMC12470433; doi:10.3390/insects16090977)
Supplement: Supplementary file 1 [file insects-16-00977-s001.zip › insects-3810329-supplementary.pdf]

**Table S1.** Calculated proximate composition of the experimental substrates (% as fed-basis).

| | SD0 | SD25 | SD50 | SD75 | SD100 |
|---|---|---|---|---|---|
| | | | *Proximate composition* | | |
| Moisture | 84.8 | 81.0 | 77.3 | 73.5 | 69.7 |
| Crude protein | 4.6 | 7.4 | 10.2 | 13.1 | 15.9 |
| Crude lipid | 5.1 | 7.7 | 10.3 | 12.8 | 15.4 |
| Ash | 1.7 | 2.1 | 2.6 | 3.1 | 3.5 |
| Crude fiber | 3.5 | 2.7 | 1.8 | 1.0 | 0.2 |

BSFL, Black soldier fly larvae; SD, sardine

SD0, SD25, SD50, SD75, and SD100 represent substrates with 0, 25, 50, 75, and 100% SD inclusion in FW-based substrates, respectively.

**Table S2.** Alpha diversity of intestinal bacterial communities of Black soldier fly larvae

| Experimental groups | Observed features | Faith pd | Chao1 | Evenness | Shannon |
|---|---|---|---|---|---|
| SD0 | 79±5.20 | 12.78±3.34 | 80.67±6.81 | 0.44±0.04 | 2.79±0.29 |
| SD25 | 88.33±22.03 | 13.74±4.65 | 88.67±22.01 | 0.44±0.03 | 2.85±0.11 |
| SD100 | 88±15.39 | 16.1±1.90 | 88±15.39 | 0.48±0.07 | 3.07±0.49 |
| p-value | 0.510 | 0.302 | 0.574 | 0.460 | 0.355 |

Values are the mean of triplicate groups and presented as mean ± SE. The absence of superscript letters indicates no significant differences among treatments.

BSFL, Black soldier fly larvae; SD, sardine

SD0, SD25, SD50, SD75, and SD100 represent substrates with 0, 25, 50, 75, and 100% SD inclusion in FW-based substrates, respectively.
